# Effects of Comprehensive Rehabilitation on Pulmonary Function in Patients Recovering from COVID-19

**DOI:** 10.3390/ijerph20053985

**Published:** 2023-02-23

**Authors:** Alicja Mińko, Agnieszka Turoń-Skrzypińska, Aleksandra Rył, Aleksandra Szylińska, Iwona Denisewicz, Iwona Rotter

**Affiliations:** 1 Department and Unit of Medical Rehabilitation and Clinical Physiotherapy, Pomeranian Medical University, 71-210 Szczecin, Poland; 2 Saint Charles Borromeo Rehabilitation Hospital, 71-899 Szczecin, Poland

**Keywords:** COVID-19, SARS-CoV-2, spirometry, rehabilitation

## Abstract

The aim of this study is to evaluate the effect of inpatient rehabilitation on pulmonary function in patients recovering from COVID-19, a multifaceted disease caused by the SARS-CoV-2 virus. This aspect of recovery is crucial, as pneumonia associated with this disease can cause lung-function abnormalities with varying degrees of hypoxemia. This study involved 150 patients qualified for inpatient rehabilitation following SARS-CoV-2 infection. Functional assessment of the lungs was performed by spirometry. The mean age of patients was 64.66 (±11.93) years, and the mean body mass index (BMI) was 29.16 (±5.68). The tests showed a statistically significant improvement in spirometric parameters. The rehabilitation program based on aerobic, strength, and endurance exercises improved lung-function parameters in the long term. The improvement in spirometric parameters in patients after COVID-19 may be related to body mass index (BMI).

## 1. Introduction

COVID-19 is a multifaceted disease caused by the SARS-CoV-2 virus that affects the respiratory, nervous, cardiovascular, and gastrointestinal systems. It was declared a pandemic by the World Health Organization (WHO) in March 2020 [1,2,3].

The lungs are the most common target of SARS-CoV-2 infection. COVID-19-associated pneumonia may cause lung-function abnormalities with varying degrees of hypoxemia, resulting in cough and shortness of breath. Chest computed tomography (CT) and chest radiography show characteristic multifocal, bilateral, patchy opacities or consolidation with interlobular septa and vascular thickening in peripheral lung areas [4,5]. Approximately 15–30% of hospitalized COVID-19 patients develop severe respiratory failure and acute respiratory distress syndrome (ARDS), which requires admission to the intensive-care unit (ICU) and mechanical ventilation [6,7,8].

About half of patients recovering from COVID-19 report chronic dyspnea months after the onset of the disease. Respiratory function testing, including spirometry, shows reduced vital capacity (FVC) and total lung capacity (TLC) in COVID-19 patients. Other symptoms include reduced diffusing capacity, lower exercise capacity, and impaired respiratory-muscle strength [9,10,11]. Risk of poor prognosis is increased by advanced age, severe pneumonia, prolonged ICU stay, and smoking [12].

Cardiopulmonary rehabilitation is the basis for dealing with people suffering from chronic lung diseases. Its strategy is based on individualized rehabilitation programs, which include aerobic exercises, breathing exercises, and muscle-strengthening exercises. There is ample evidence supporting the benefits of pulmonary rehabilitation in terms of disease mortality, improved cardiorespiratory fitness, and quality of life [13,14].

Improved respiratory function is an important symptom of recovery from COVID-19. According to the recommendations of scientific societies, pulmonary rehabilitation programs should be implemented in patients after COVID-19 [15,16,17,18]. Research shows that pulmonary rehabilitation programs in patients after COVID-19 positively affect the improvement of lung-function parameters, such as forced expiratory volume in one second (FEV1), forced vital capacity (FVC), and the Tiffeneau-Pinelli index [19]. Other studies show that an eight-week rehabilitation program consisting of aerobic and resistance exercises significantly improved cardiopulmonary and skeletal-muscle functions in COVID-19 patients [20]. The authors recommend the use of respiratory-muscle training with resistance, effective cough exercises, and respiratory-muscle stretching exercises in respiratory rehabilitation programs for patients after COVID-19. According to the literature, physiotherapy after COVID-19 should also include aerobic and/or resistance training [14,18,19,20,21].

The aim of this study is to evaluate the effect of inpatient rehabilitation, implemented according to the Polish National Health Fund guidelines, on lung function in COVID-19 patients. The effectiveness of rehabilitation was measured by the improvement in spirometric parameters. An additional objective is to determine the relationship between spirometric parameters before and after rehabilitation and gender, age, body mass index (BMI), length of hospitalization, and the presence of COVID-19-associated pneumonia.

## 2. Materials and Methods

The study was conducted between May 2021 and September 2022 at the St. Charles Borromeo Rehabilitation Hospital in Szczecin (Poland), where inpatient rehabilitation of COVID-19 patients was carried out in accordance with the guidelines of the National Health Fund (NFZ), as indicated in Order No. 42/2021/DSOZ of the President of the NFZ dated 5 March 2021.

### 2.1. Characteristics of the Study Group

The study included 171 patients qualified for inpatient rehabilitation following SARS-CoV-2 infection, each based on a referral issued by a physician no later than 12 months after the completion of treatment for COVID-19, confirmed by a positive polymeraze chain reaction (PCR) test for SARS-CoV-2. The completion of treatment was defined as the date of completion of home isolation, discharge from the hospital, or end of isolation.

Patients’ eligibility criteria were post-COVID-19 respiratory or cardiovascular or nervous-system or musculoskeletal complications (with a score of 1–4 when assessed on a scale of 0 to 4 based on the Post-COVID-19 Functional Status (PCFS) Scale). Patients who experienced a decrease in muscle strength (as defined by the Medical Research Council (MRC) muscle-strength score) or an increase in dyspnea (with a score ≥ 1 when assessed on a 0 to 4 scale based on the modified Medical Research Council (mMRC) dyspnea score) were also eligible for rehabilitation. Only patients who gave informed consent were included in the study.

**Figure 1:**
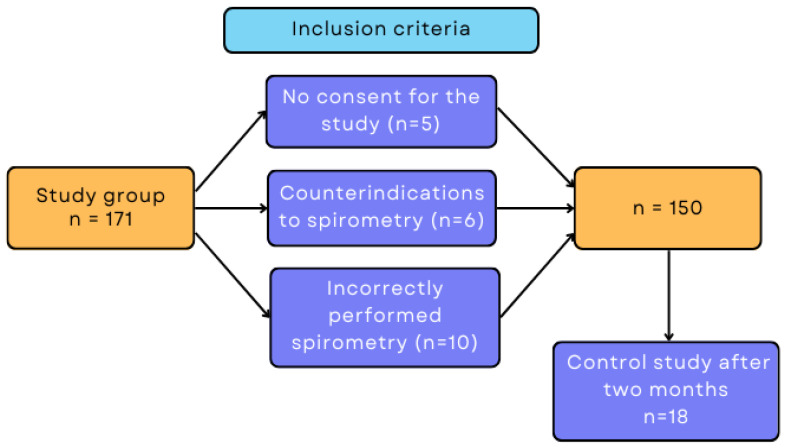
Flowchart of inclusion and exclusion criteria.

The exclusion criteria for the study were age under 18 and lack of consent. Patients who performed the spirometric test incorrectly or could not perform it due to contraindications were excluded from the study. Taking into account all inclusion and exclusion criteria, 150 patients participated in the study (Figure 1).

### 2.2. Course of the Study

Patients actively participated in the post-COVID-19 rehabilitation program, according to the regimen of the St. Charles Borromeo Rehabilitation Hospital in Szczecin. Rehabilitation activities were carried out six times a week, from Monday to Saturday. Sunday was a day off. A detailed rehabilitation program is presented in Table 1.

The duration of rehabilitation, as recommended by the National Health Service, lasted 2 to 6 weeks. Throughout the rehabilitation period, each patient was under medical and nursing care. The length of the patient’s stay in the rehabilitation unit was decided by the attending physician, based on a comparison of the results of examinations and tests, such as the exercise test with exercise tolerance assessment and spirometric respiratory functional assessment, carried out at the beginning and end of treatment.

Functional assessment of the lungs was determined by spirometry on the day of admission to the rehabilitation unit, on the day of discharge from the rehabilitation unit, and 2 months after completion of rehabilitation. The portable spirometer BTL-08 Spiro (BTL, Newcastle-under-Lyme, UK) was used for the measurement. The following parameters were determined: forced expiratory volume in the first second (FEV 1), forced vital capacity (FVC), FEV 1/FVC ratio, maximal expiratory flow (MEF) 25–75%, maximal mid-expiratory flow (MMEF), and peak expiratory flow (PEF). Results were expressed as a percentage of the patient’s predicted normal values, which were calculated automatically based on age, gender, height, weight, and ethnicity. The FEV1/FVC ratio is shown as an absolute value. For the interpretation of spirometry data, ECCS/ERS 1993 reference values were used. Normal values for these parameters are FVC > 80%, FEV1 > 75%, FEV1/FVC > 75%, MEF75, MEF50, and MEF25 > 75% of the reference value [22]. All spirometric measurements were performed in accordance with standard American Thoracic Society (ATS) and European Respiratory Society (ERS) recommendations [23].

On the day of admission to the rehabilitation department, an additional interview was conducted to obtain demographic data. Information regarding the patient’s medical history and treatment, as well as comorbidities, was obtained from medical records.

The study was approved by the Bioethics Committee of Pomeranian Medical University in Szczecin (decision no. KB-0012/15/2021).

### 2.3. Statistical Methods

Statistical analysis was carried out using Statistica (version 13.1). Characterization of the study group was performed, taking into account the number of patients, patients’ percentage, mean, median, first and third quartiles, and standard deviation. Normality of distribution was tested using the Shapiro–Wilk test. For the normal distribution, the data are presented in the mean and standard deviation. For the non-normal distribution, the data are presented in the median, first, and third quartiles. The differences between two groups were analyzed using Student’s *t*-test and the Mann–Whitney U-test. The difference between multiple groups was analyzed by the Kruskal–Wallis test or by the ANOVA test. Correlation analysis was performed using Spearman’s Rho test. Nominal variables were tested using the chi-squared test. Dependent variables were tested with the *t*-test for dependent samples and the Wilcoxon test. Multivariable logistic regression analysis was performed. For the logistic regression analysis, an increase in FVC after rehabilitation was assumed as a predictor of lung-function improvement. The model for multivariable logistic regression was adjusted for gender, age, body mass index, pneumonia, and length of hospitalization. A *p*-value of <0.05 was regarded as statistically significant.

## 3. Results

### 3.1. Group Characteristics

A total of 150 subjects were included in the study. They were characterized in terms of age, gender, and nutritional status. The need for hospitalization, length of hospitalization, presence of pneumonia in the course of SARS-CoV-2 infection, and length of stay in the rehabilitation unit after COVID-19 were also analyzed.

The average age in the study group was 64.66 (±11.93) years. The average height and weight of the study population were 168.08 cm (±9.5) and 82.67 kg (±17.63), respectively. The average body mass index (BMI) was 29.16 (±5.68). The characteristics of the study population are shown in Table 2.

### 3.2. Spirometric Results before and after Rehabilitation

The analysis of the results is presented in the form of correlations between spirometric test parameters before and after rehabilitation. Improvement in spirometric parameters was observed for FVC (*p* = 0.016), FEV1 (*p* < 0.001), PEF (*p* < 0.001), MMEF (*p* < 0.001), MEF75 (*p* < 0.001), and MEF 50 (*p* < 0.001). Detailed results are shown in Table 3.

### 3.3. Spirometric Results Immediately after Rehabilitation and after 2 Months

Table 4 shows correlations between spirometric test parameters after and 2 months after rehabilitation. There were no significant differences between the variables studied.

### 3.4. Difference in Spirometric Results before and after Rehabilitation vs. Selected Variables

Table 5 shows the relationships between the difference in spirometric results before and after rehabilitation and gender. According to the analysis, gender did not affect the results.

Table 6 shows the relationship between the difference in spirometric results before and after rehabilitation and the presence of COVID-19 pneumonia. There was no difference in results between subjects without and with COVID-19 pneumonia.

Table 7 shows the relationship between the difference in spirometric results before and after rehabilitation and the degree of obesity. According to the analysis, people with a higher degree of obesity obtained less improvement in parameters such as FVC (*p* = 0.026) and FEV1 (*p* = 0.007).

Table 8 shows the relationships between the difference in spirometric results before and after rehabilitation and age. The age of the subjects was not significant in relation to the results obtained.

Table 9 shows the correlations between the difference in spirometric results before and after rehabilitation and the length of hospitalization during COVID-19 infection. Statistical significance was related to parameters such as FEV1/FVC (*p* = 0.033).

A post hoc analysis of statistically significant differences in parameters in the intergroup analysis of multiple variables (degree of obesity vs. FVC, degree of obesity vs. FEV1, and length of hospitalization vs. FEV1/FVC) was performed. It was shown that there were no statistically significant differences between the analyzed groups.

**Table 7:** Relationships between the difference in spirometric results before and after rehabilitation and BMI.

Variable		Correct Weight (n = 30)	Overweight (n = 53)	1st Degree Obesity (n = 46)	2nd Degree Obesity (n = 15)	3rd Degree Obesity (n = 5)	*p*
**Δ FVC** (% predicted)	Me (Q1–Q3)	10.86 (7.7–20.1)	4.1 (−1.6–13.06)	3.1 (−5.5–13.15)	11.8 (2.7–15.9)	3.2 (−3.8–7.3)	0.026 *
**Δ FEV1** (% predicted)	M ± SD	12.35 ± 13.76	10.87 ± 6.99	6.85 ± 16.25	12.75 ± 15.24	−11.99 ± 13.66	0.007 *
**Δ FEV1/FVC** (%)	Me (Q1–Q3)	−1.25 (−5.8–6.6)	3.27 (−4.6–12.6)	0.7 (−3.7–8.76)	0.04 (−8.4–10.6)	0.3 (−5.2–4.9)	0.712
**Δ PEF** (% predicted)	M ± SD	20.15 ± 19.31	24.98 ± 24.66	18.24 ± 22.32	19.73 ± 26.09	18.02 ± 15.43	0.428
**Δ MMEF** (% predicted)	M ± SD	15.38 ± 27.59	19.43 ± 16.9	14.25 ± 30.94	14.47 ± 32.26	−7.98 ± 13.85	0.348
**Δ MEF75** (% predicted)	Me (Q1–Q3)	24.27 (8.02–35.8)	28.2 (12.1–42.1)	13.6 (1.28–38.8)	24.6 (6.3–42.01)	23.3 (5–29.5)	0.461
**Δ MEF50** (% predicted)	Me (Q1–Q3)	21.1 (0.24–35.2)	15.3 (−0.8–35.3)	6.5 (−4.4–43.8)	23.1 (−10.4–45.7)	3.54 (−15–8.3)	0.456
**Δ MEF25** (% predicted)	M ± SD	−5.86 ± 41.98	9.98 ± 5.46	2.38 ± 48.61	−5.56 ± 41.47	−28.61 ± 15.32	0.188

Legend: *p*—statistical significance *, M—mean, SD—standard deviation, Me—median, Q1—first quartile, Q3—third quartile, n—number, Δ—spirometry final value minus baseline value.

**Table 8:** Relationships between the difference in spirometric results before and after rehabilitation and age.

Variable		30–45 Years (n = 12)	46–60 Years (n = 31)	61–75 Years (n = 84)	76–90 Years (n = 23)	*p*
**Δ FVC** (% predicted)	Me (Q1–Q3)	7.05 (−0.24–19.6)	8.6 (2.3–16.7)	6.2 (−3.9–12.6)	7.47 (−0.26–16.6)	0.562
**Δ FEV1** (% predicted)	M ± SD	11.63 ± 9.76	10.12 ± 18.41	8.76 ± 15.41	9.91 ± 14.91	0.711
**Δ FEV1/FVC** (%)	Me (Q1–Q3)	3.2 (−5.04–7.5)	−0.8 (−6.3–6.65)	2.15 (−4.8–9.7)	0.3 (−8.9–17.34)	0.837
**Δ PEF** (% predicted)	M ± SD	23.33 ± 17.36	14.61 ± 21.03	24.35 ± 21.71	17.15 ± 16.23	0.141
**Δ MMEF** (% predicted)	Me (Q1–Q3)	9.6 (−4.25–30.3)	7.23 (−11.2–24.7)	17.6 (−5.25–38.9)	3.7 (−10.8–29.5)	0.378
**Δ MEF75** (% predicted)	Me (Q1–Q3)	27.1 (11.7–44.7)	15.75 (1.9–35.8)	26.65 (7.2–43.01)	17.4 (4.9–32.3)	0.322
**Δ MEF50** (% predicted)	Me (Q1–Q3)	11.4 (3.6–20.5)	12.8 (−6.03–23.7)	15.3 (−1.4–44.3)	7.9 (−6.4–35.7)	0.556
**Δ MEF25** (% predicted)	Me (Q1–Q3)	10.43 (−7.25–27.3)	−3.3 (−26.7–19.1)	−2.5 (−28–28.6)	−1.7 (−42.8–41.7)	0.846

Legend: *p*—statistical significance, M—mean, SD—standard deviation, Me—median, Q1—first quartile, Q3—third quartile, n—number, Δ—spirometry final value minus baseline value.

**Table 9:** Relationships between the difference in spirometric results before and after rehabilitation and the length of hospitalization in the course of COVID-19.

Variable		1–5 Days (n = 5)	6–10 Days (n = 12)	11–15 Days (n = 28)	16–20 Days (n = 15)	Over 20 Days (n = 48)	*p*
		M ± SD	M ± SD	M ± SD	M ± SD	M ± SD	
**Δ FVC** (% predicted)	Me (Q1–Q3)	5.46 (2.6–19.5)	3.65 (−7.6–13.7)	3.4 (−4.3–16.13)	6.06 (−1.6–10.6)	11 (−0.74–14.1)	0.659
**Δ FEV1** (% predicted)	Me (Q1–Q3)	6.6 (4.5–10.4)	13.15 (−3.6–22.9)	3.2 (−2.06–18.02)	2.5 (0.7–11.5)	12.02 (4.3–22.4)	0.231
**Δ FEV1/FVC** (%)	Me (Q1–Q3)	1.6 (−27.9–4.9)	5.7 (−1.4–20.6)	1.38 (−3.7–12.8)	−1.15 (−8.9–3.7)	1.94 (−5.3–8.9)	0.033 *
**Δ PEF** (% predicted)	Me (Q1–Q3)	19.3 (10.3–27)	18.9 (8.2–26.3)	25.3 (8.2–32.5)	15.3 (2.6–27)	33.4 (15.7–43.6)	0.174
**Δ MMEF** (% predicted)	Me (Q1–Q3)	11.9 (−1.6–18.4)	16.4 (2.8–40.8)	14.7 (−9.2–36.3)	0.14 (−8.1–17.3)	23 (−4.9–46.2)	0.355
**Δ MEF75** (% predicted)	M ± SD	51.21 ± 71.49	21.19 ± 18.85	20.18 ± 20.94	15.75 ± 19.07	32.05 ± 25.11	0.057
**Δ MEF50** (% predicted)	Me (Q1–Q3)	20.9 (2.7–30.3)	11.6 (0.9–37.2)	14.04 (−3–34.6)	0.71 (−12.8–22.1)	23.3 (2.17–50.2)	0.171
**Δ MEF25** (% predicted)	Me (Q1–Q3)	−25.11 (−29.5- −2.8)	22.6 (6.9–43.4)	0.01 (−33.5–21.6)	−21.8 (−32.4–4.08)	13.6 (−22.2–34.3)	0.068

Legend: *p*—statistical significance *, M—mean, SD—standard deviation, Me—median, Q1—first quartile, Q3—third quartile, n—number, Δ—spirometry final value minus baseline value.

The results of the multivariate regression analysis are presented in Table 10. The analysis showed a significant decrease in the improvement of the lung function in patients with overweight (OR = 0.176, *p* = 0.012) and first-degree obesity (OR = 0.117, *p* = 0.002).

## 4. Discussion

Cardiopulmonary rehabilitation programs are recommended in the care of patients recovering from COVID-19 to prevent long-term complications in the musculoskeletal, respiratory, and circulatory systems. Individualized rehabilitation programs that include aerobic, muscle-strengthening, and respiratory exercises may allow a faster recovery of COVID-19 patients [19,24].

The present study evaluated the short- and long-term effects of rehabilitation on spirometric parameters in COVID-19 patients. A multivariate analysis was also performed to assess the relationship between spirometric parameters before and after rehabilitation and gender, age, body mass index (BMI), length of hospitalization, and the presence of SARS-CoV-2 infection pneumonia.

The present study showed a significant improvement in pulmonary function confirmed by spirometry after rehabilitation compared to the pre-rehabilitation evaluation. Similar results are presented by Hockele et al. [25], who used a similar rehabilitation regimen with a different frequency of exercise. The authors used a regimen consisting of respiratory, aerobic, and resistance exercises performed twice a week for 60 min each. The study by Douin et al. [26] used a cardiopulmonary rehabilitation program consisting of respiratory exercises, dynamic exercises based on interval training, and resistance exercises for major muscle groups. The authors also used a different frequency of training sessions. Sessions were held three times a week for a maximum of 1.5 h. Despite significant differences in the volume of exercises performed, patients achieved significant improvements in spirometric parameters.

Our study was not randomized and did not include a control group for those with COVID-19 but without rehabilitation. Following the study of Liu et al. [19], who included a control group in their study, we can conclude that the results obtained were comparable. After 6 weeks of pulmonary rehabilitation, there was a statistically significant difference between FEV1, FVC, and FEV1/FVC when comparing the study group with the control group.

A number of authors evaluated the lung function of COVID-19 patients at different times since recovery but without any rehabilitation [27,28,29]. Zhao et al. [30] showed persistent impairment of FEV1 and FEV1/FVC in about 32.42% of cases three months after recovery from COVID-19. Liang et al. [31] showed persistent abnormalities in lung function, consisting of a decrease in FVC 6 weeks after hospital discharge. This indicates that early rehabilitation interventions seem to be an essential element in recovery from COVID-19.

To the best of our knowledge, the present study is the first to evaluate the factors affecting the improvement in spirometric parameters in patients after COVID-19. Our study shows that people with overweight and the first-degree of obesity showed significantly less improvement in lung-function parameters after rehabilitation. Guler et al. [28] showed that obesity was associated with lower spirometric results in patients after COVID-19. However, their study did not take into account the difference in the parameters studied before and after rehabilitation.

A limitation of this study was the lack of a control group. However, for ethical reasons, this was not possible. Patients with asthma and COPD who may have already had pulmonary dysfunction were included in this study. However, after analyzing the results of the spirometry test, they showed no significant differences from the rest of the group. Another limitation of this study was the varying time between the onset of COVID-19 and the first day of rehabilitation. However, it was not longer than 12 months. The duration of the rehabilitation cycle was also not uniform—it ranged from 2 to 6 weeks. The indicated factors may affect the final results of this study.

The strength of this study is the supervised rehabilitation process. All patients performed exercises in a stationary center under the constant supervision of physiotherapists. In future studies, it would be interesting to investigate respiratory function with additional tests, such as the plethysmography or lung gas diffusion capacity (DLCO) tests.

## 5. Conclusions

A rehabilitation program based on aerobic, strength, and endurance exercises improved lung-function parameters in patients after their recovery from COVID-19. The achieved effects of rehabilitation were sustained in the long term. The improvement in spirometric parameters in patients after COVID-19 may be related to body mass index (BMI).

## Figures and Tables

**Table 1:** Rehabilitation program.

Procedure	Guidelines	Time
General fitness and respiratory improvement exercises	Active breathing exercises, active breathing exercises with resistance, learning to cough and expectorate effectively	30 min
Aerobic training	Stair climbing; climbing 2–3 floors at a leisurely pace; gradually increased intensity by 5–10%; assessment of exercise tolerance by oxygenation checks (pulse oximeter); perceived exercise intensity: 2–3 on the Borg scale	30 min
Continuous/interval endurance training on a bicycle cycloergometer	Initial low or moderate intensity; gradual increase in intensity by 5–10%; assessment of exercise tolerance by oxygenation checks (pulse oximeter); perceived exercise intensity: 2–3 on the Borg scale	30 min
Outdoor march training	Assessment of exercise tolerance by oxygenation checks (pulse oximeter); perceived of exercise intensity: 2–3 on the Borg scale	2 times a day for 30 min each
Strength and endurance training	Training selected individually for the patient on the basis of the RM unit and the patient’s exercise tolerance (assessment of the presence of desaturation); load: 70–85% of 1 RM; volume: 3 series of 8–12 repetitions; rest: 1–2 min; progression: 60–70% of 1 RM	30 min

Legend: RM (repetition maximum) the maximum number of repetitions in a series possible with a given load; 1 RM (one repetition max) is the maximum single repetition with maximum load.

**Table 2:** Characteristics of the study group.

Variable		n	%
Gender	female	94	54.97
	male	77	45.03
Age	30–45 years	15	8.77
	46–60 years	33	19.3
	61–75 years	96	56.14
	76–90 years	27	15.79
Nutritional status (BMI)	18.5–24.99 (norm)	37	21.76
	25.0–29.9 (overweight)	61	35.88
	30.0–34.99 (1st degree obesity)	49	28.82
	35.0–39.99 (2nd degree obesity)	18	10.59
	over 40 (3rd degree obesity)	5	2.94
Hospitalization	yes	119	69.59
	no	45	26.32
Length of hospitalization	1–5 days	5	2.92
	6–10 days	13	7.6
	11–15 days	32	18.71
	16–20 days	18	10.53
	over 20 days	51	29.82
Pneumonia in the course of COVID-19	yes	127	74.27
	no	37	21.64
Duration of rehabilitation	2–3 weeks	80	46.78
	3–4 weeks	27	15.79
	4–5 weeks	23	13.45
	5–6 weeks	41	23.97
Comorbidities	diabetes	43	25.15
	hypertension	97	56.73
	asthma	19	11.11
	COPD	8	4.68

Legend: n—number, BMI—body mass index, COPD—chronic obstructive pulmonary disease.

**Table 3:** Relationships between spirometric results before and after rehabilitation.

Variable		Before Rehabilitation (n = 150)	After Rehabilitation (n = 150)	*p*
**FVC** (% predicted)	M ± SD	83.39 ± 22.71	89.71 ± 21.47	0.016 *
**FEV1** (% predicted)	Me (Q1–Q3)	86.36 (68.16–102.75)	96.81 (80.73–110.43)	<0.001 *
**FEV1/FVC** (%)	Me (Q1–Q3)	86.32 (78.52–91.51)	87.39 (81.46–91.93)	0.071
**PEF** (% predicted)	Me (Q1–Q3)	56.5 (36.85–76.05)	82.13 (60.12–99.36)	<0.001 *
**MMEF** (% predicted)	Me (Q1–Q3)	82.35 (60.12–108.03)	99.38 (77.15–125.43)	<0.001 *
**MEF75** (% predicted)	Me (Q1–Q3)	53.48 (36.27–81.33)	85.15 (59.78–106.08)	<0.001 *
**MEF50** (% predicted)	Me (Q1–Q3)	73.97 (51.62–102.73)	95.81 (70.25–119.86)	<0.001 *
**MEF25** (% predicted)	Me (Q1–Q3)	106.76 (75.77–141.97)	108.3 (80.67–146.97)	0.554

Legend: *p*—statistical significance *, M—mean, SD—standard deviation, Me—median, Q1—first quartile, Q3—third quartile, n—number.

**Table 4:** Relationships between spirometric results after and 2 months after rehabilitation.

Variable		After Rehabilitation (n = 150)	2 Months after Rehabilitation (n = 18)	*p*
		M ± SD	M ± SD	
**FVC** (% predicted)	M ± SD	89.71 ± 21.47	83.63 ± 15.41	0.304
**FEV1** (% predicted)	M ± SD	94.71 ± 22.69	90.11 ± 15.27	0.265
**FEV1/FVC** (%)	Me (Q1–Q3)	87.39 (81.46–91.93)	85.35 (83.44–92.03)	0.084
**PEF** (% predicted)	M ± SD	80.24 ± 27.98	92.04 ± 33.26	0.235
**MMEF** (% predicted)	M ± SD	101.47 ± 39.6	103.22 ± 38.43	0.814
**MEF75** (% predicted)	M ± SD	84.37 ± 31.65	89.94 ± 35.04	0.781
**MEF50** (% predicted)	M ± SD	95.69 ± 37.35	94.53 ± 35.53	0.934
**MEF25** (% predicted)	Me (Q1–Q3)	108.3 (80.67–146.97)	108.77 (89.04–119.05)	0.915

Legend: *p*—statistical significance, M—mean, SD—standard deviation, Me—median, Q1—first quartile, Q3—third quartile, n—number.

**Table 5:** Relationships between the difference in spirometric results before and after rehabilitation and gender.

Variable		Female (n = 85)	Male (n = 65)	*p*
**Δ FVC** (% predicted)	M ± SD	6.56 ± 14.64	6.34 ± 13.45	0.601
**Δ FEV1** (% predicted)	Me (Q1–Q3)	5.86 (0.58–18.11)	10.33 (1.52–16.23)	0.288
**Δ FEV1/FVC** (%)	Me (Q1–Q3)	0.3 (−5.4–8.67)	2.71 (−3.68–12.57)	0.562
**Δ PEF** (% predicted)	M ± SD	18.95 ± 21.12	24.04 ± 20.04	0.123
**Δ MMEF** (% predicted)	Me (Q1–Q3)	9.07 (−7.91–29.55)	16.14 (−5.78–36.56)	0.509
**Δ MEF75** (% predicted)	Me (Q1–Q3)	23.13 (4.56–38.76)	24.61 (9.16–40.74)	0.366
**Δ MEF50** (% predicted)	Me (Q1–Q3)	11.4 (−4.48–32.35)	13.46 (−0.87–37.77)	0.411
**Δ MEF25** (% predicted)	M ± SD	−1.5 ± 46.75	6.05 ± 46.56	0.192

Legend: *p*—statistical significance, M—mean, SD—standard deviation, Me—median, Q1—first quartile, Q3—third quartile, n—number, Δ—spirometry final value minus baseline value.

**Table 6:** Relationships between the difference in spirometric results before and after rehabilitation and the presence of COVID-19 pneumonia.

Variable		Pneumonia (n = 115)	No Pneumonia (n = 32)	*p*
**Δ FVC** (% predicted)	M ± SD	6.66 ± 13.51	5.62 ± 16.71	0.549
**Δ FEV1** (% predicted)	Me (Q1–Q3)	6.96 (0.5–17.91)	5.03 (1.41–13.36)	0.522
**Δ FEV1/FVC** (%)	Me (Q1–Q3)	1.76 (−5.17–8.9)	−0.62 (−7.08–13.29)	0.604
**Δ PEF** (% predicted)	Me (Q1–Q3)	22.88 (9.17–35.9)	11.64 (−1.97–30.35)	0.079
**Δ MMEF** (% predicted)	Me (Q1–Q3)	14.69 (−4.62–34.67)	−2.28 (−11.06–24.97)	0.069
**Δ MEF75** (% predicted)	Me (Q1–Q3)	23.46 (6.42–40.74)	20.75 (−2.34–34.6)	0.221
**Δ MEF50** (% predicted)	Me (Q1–Q3)	15.33 (−1.57–39.04)	2.92 (−5.74–23.48)	0.099
**Δ MEF25** (% predicted)	Me (Q1–Q3)	0.44 (−26.69–27.72)	−9.89 (−42.03–12.87)	0.148

Legend: *p*—statistical significance M—mean, SD—standard deviation, Me—median, Q1—first quartile, Q3—third quartile, n—number, Δ—spirometry final value minus baseline value.

**Table 10:** Multivariate logistic regression for predicting lung-function improvement after rehabilitation.

Outcome		OR (95% CI)	*p*
Gender	male	1.190 (0.550–2.574)	0.659
Age	30–45 years	1.000 (-)	-
	46–60 years	1.446 (0.272–7.696)	0.665
	61–75 years	0.644 (0.150–2.776)	0.555
	76–90 years	0.969 (0.184–5.108)	0.970
Nutritional status (BMI)	norm	1.000 (-)	-
	overweight	0.176 (0.046–0.678)	0.012 *
	1st degree obesity	0.117 (0.030–0.452)	0.002 *
	2nd degree obesity	0.619 (0.088–4.340)	0.629
	3rd degree obesity	0.155 (0.018–1.377)	0.094
Hospitalization		1.405 (0.431–4.581)	0.573
Pneumonia in the course of COVID-19		0.968 (0.271–3.462)	0.961

Legend: *p*—statistical significance *, OR—odds ratio, CI—confidence interval.

## Data Availability

All data was collected in the Department and Unit of Medical Rehabilitation and Clinical Physiotherapy, Pomeranian Medical University, 71-210 Szczecin.
